# Relationships between trace elements and cognitive and depressive behaviors in sprague dawley and wistar albino rats

**DOI:** 10.3389/fphar.2024.1367469

**Published:** 2024-04-02

**Authors:** Melis Yavuz, Ekin Dongel Dayanc, Fatma Merve Antmen, Elif Keskinöz, Esra Altuntaş, Gökçen Dolu, Berkcan Koç, Emre Tunçcan, Damla Şakar, Ufuk Canözer, Ceyda Büyüker, Ece Polat, Metincan Erkaya, Rui Azevedo, Devrim Öz Arslan, Agostinho Almeida, Güldal Süyen

**Affiliations:** ^1^ Department of Pharmacology, Faculty of Pharmacy, Acibadem Mehmet Ali Aydinlar University, Istanbul, Türkiye; ^2^ Department of Physiology, Institute of Health Sciences, Acibadem Mehmet Ali Aydinlar University, Istanbul, Türkiye; ^3^ Department of Medical Laboratory Techniques, Vocational School of Health Services, Acibadem Mehmet Ali Aydinlar University, Istanbul, Türkiye; ^4^ Biobank Unit, Acibadem Mehmet Ali Aydinlar University, Istanbul, Türkiye; ^5^ Department of Anatomy, School of Medicine, Acibadem Mehmet Ali Aydinlar University, Istanbul, Türkiye; ^6^ Faculty of Pharmacy, Acibadem Mehmet Ali Aydinlar University, Istanbul, Türkiye; ^7^ Department of Biophysics, Institute of Health Sciences, Acibadem Mehmet Ali Aydinlar University, Istanbul, Türkiye; ^8^ School of Medicine, Acibadem Mehmet Ali Aydinlar University, Istanbul, Türkiye; ^9^ LAQV/REQUIMTE, Department of Chemical Sciences, Faculty of Pharmacy, University of Porto, Porto, Portugal; ^10^ Department of Biophysics, School of Medicine, Acibadem Mehmet Ali Aydinlar University, Istanbul, Türkiye; ^11^ Department of Physiology, School of Medicine, Acibadem Mehmet Ali Aydinlar University, Istanbul, Türkiye

**Keywords:** social isolation, memory, Strain, sprague Dawley, Wistar, middle-aged, trace elements, depression

## Abstract

**Introduction:** This study investigates the effects of social isolation on mental health and cognitive functions in Sprague Dawley (SD) and Wistar Albino (WIS) rat strains, prompted by the heightened awareness of such impacts amid the COVID-19 pandemic. This study aims to explore the impact of social isolation on memory, learning, and behavioral changes in middle-aged SD and WIS rat strains and to investigate cortical trace element levels, seeking potential correlations between these levels and the observed behavioral responses to social isolation.

**Methods:** Four groups of 14-month-old male rats were established: control and isolated SDs and WIS rats (CONT-SD, ISO-SD, CONT-WIS, ISO-WIS). Morris Water Maze and Porsolt Forced Swimming tests were conducted for behavioral assessment. Following behavioral tests, rats were sacrificed under general anesthesia, and cortices were isolated for analysis of macro and trace element levels (ICP/MS).

**Results:** In behavioral tests, CONT-SD rats exhibited superior performance in the Morris Water Maze test compared to CONT-WIS rats, but displayed increased depressive behaviors following social isolation, as evident in the Porsolt Forced Swimming test (*p* < 0.05). ISO-SD rats showed elevated levels of Co and Cu, along with reduced levels of Cs and As, compared to ISO-WIS rats. Moreover, isolation resulted in decreased Cu and Mo levels but increased Rb levels in WIS rats. Comparison of trace element levels in naïve groups from different strains revealed lower Zn levels in the WIS group compared to SD rats.

**Discussion:** The findings suggest that the SD strain learns faster, but is more susceptible to depression after isolation compared to the WIS strain. Increased Co and Cu levels in ISO-SD align with previous findings, indicating potential trace element involvement in stress responses. Understanding these mechanisms could pave the way for preventive treatment strategies or therapeutic targets against the consequences of stressors, contributing to research and measures promoting a balanced diet to mitigate neurobehavioral abnormalities associated with social isolation in the future.

## 1 Introduction

The COVID-19 pandemic has highlighted the importance of social isolation. Therefore, understanding the effects of social isolation on behavioral and cognitive functions has been clearly felt. Social isolation is considered a potent stressor for both animals and humans (Liu et al., 2010) with significant adverse effects on the mental and cognitive health of people of all ages (Cacioppo and Hawkley, 2009). Numerous animal studies have shown that stress resulting from social isolation can cause anxiety-like behaviors and a tendency toward depression (Lukkes et al., 2009; Amiri et al., 2015). Specifically, depression and anxiety-like behavior have been shown to develop after 3 weeks of social isolation (Filipović et al., 2011; Martinovic et al., 2014). Porsolt et al. initially designed the forced swim test to assess the effectiveness of antidepressant drugs in rodents, mainly rats and mice, in their studies from 1977 to 1978 (Porsolt et al., 1977). In addition to this, this test has also been used to assess how exposure to stressful situations, which can induce depression-like behaviors, affects individuals (Armario, 2021).

Several studies have indicated long-term social isolation impairs synaptic plasticity and spatial memory (Wang et al., 2019). The Morris water maze test is a major behavioral test used to measure learning and memory function in animals. A study, assessing learning and memory functions in male albino Wistar (WIS) rats revealed that memory performance was impaired in isolated rats compared to those living in a social and enriched environment (Keloglan et al., 2022). Furthermore, social isolation increases the risk of future cognitive impairment and enhances the rate of memory decline in older people (Bassuk et al., 1999; Ertel et al., 2008). The risk of Alzheimer’s disease, which causes cognitive decline and loss of memory function, doubles in solitary individuals (Wilson et al., 2007).

Most studies on social isolation have been conducted on animals, particularly during the post-weaning, developmental or post-natal period, often as a model for autism spectrum disorders or other neurodevelopmental disorders. However, studies on middle-aged rats are rare (Flurkey et al., 2007; Flurkey and Currer, 2009; Stowie and Glass, 2015). Given that the middle-aged or elderly were mainly impacted by social isolation, which affects memory, during the pandemic, understanding the mechanisms underlying the effects of social isolation in middle-aged individuals is important, just as in adulthood.

Trace elements play crucial roles in mental illnesses (Młyniec et al., 2015; Alghadir et al., 2016). Numerous studies have shown an imbalance in the serum or plasma levels of trace and macro elements such as Zn, Cu, Fe and Mg, in individuals with depression compared to those without (Chang et al., 2014; Stanisławska et al., 2014). A study revealed that rats resistant to depression exhibited higher levels of Fe, Na, S, Mn and Co in their brain samples compared to the sensitive group. Conversely, the sensitive group displayed lower plasma levels of Ca, K, S, Se and Co compared to the resistant group (Xu et al., 2020).

In this study, we hypothesized that the prolonged social isolation experienced during the COVID-19 pandemic would exert significant effects on behavior and cognitive functions in middle-aged rats, manifesting as anxiety-like behaviors, depression, and cognitive decline in both WIS and Sprague Dawley (SD) strains. Additionally, our hypothesis posits that the protracted social isolation may lead to compromised synaptic plasticity and spatial memory, with a potential correlation to the imbalance of trace elements, particularly Zn, Cu, Fe, Mg, within the cortex. Through this study, we aim to provide insights into the underlying mechanisms of memory impairment, explore the association between trace elements and cognitive outcomes, and discern the suitability of WIS and SD rat strains for experimental investigations into the impacts of social isolation.

## 2 Materials and methods

### 2.1 Animals and housing

The rats were bred and housed in cages (n = 3 rats/cage) in the laboratory of Acıbadem Mehmet Ali Aydınlar University, Experimental Animals Application and Research Center (ACUDEHAM) (Protocol no: 2021/471). All animal experiments conformed with the EU Directive 2010/63/EU and ARRIVE guidelines. Before the experiment, the animals were subjected to a 12:12 h light-dark cycle, at a constant temperature environment (21°C ± 3°C) with 51% humidity with food and water *ad libitum*. The food provided to all animals was a standard maintenance diet for rats and mice involving constant levels of minerals and trace elements (Rats and Mice Maintenance, Carfil Quality). The rats in the social isolation group were housed individually in cages. Cotton and disposable non-transparent cardboard cylinders were placed in all cages (22 cm wide, 37 cm 18 cm high) for “enrichment”. Using 14-month-old male SD and WIS rats the experiments were performed between 10:00 a.m. and 7:00 p.m. after 4 weeks of housing.

### 2.2 Experimental design

**FIGURE 1:**
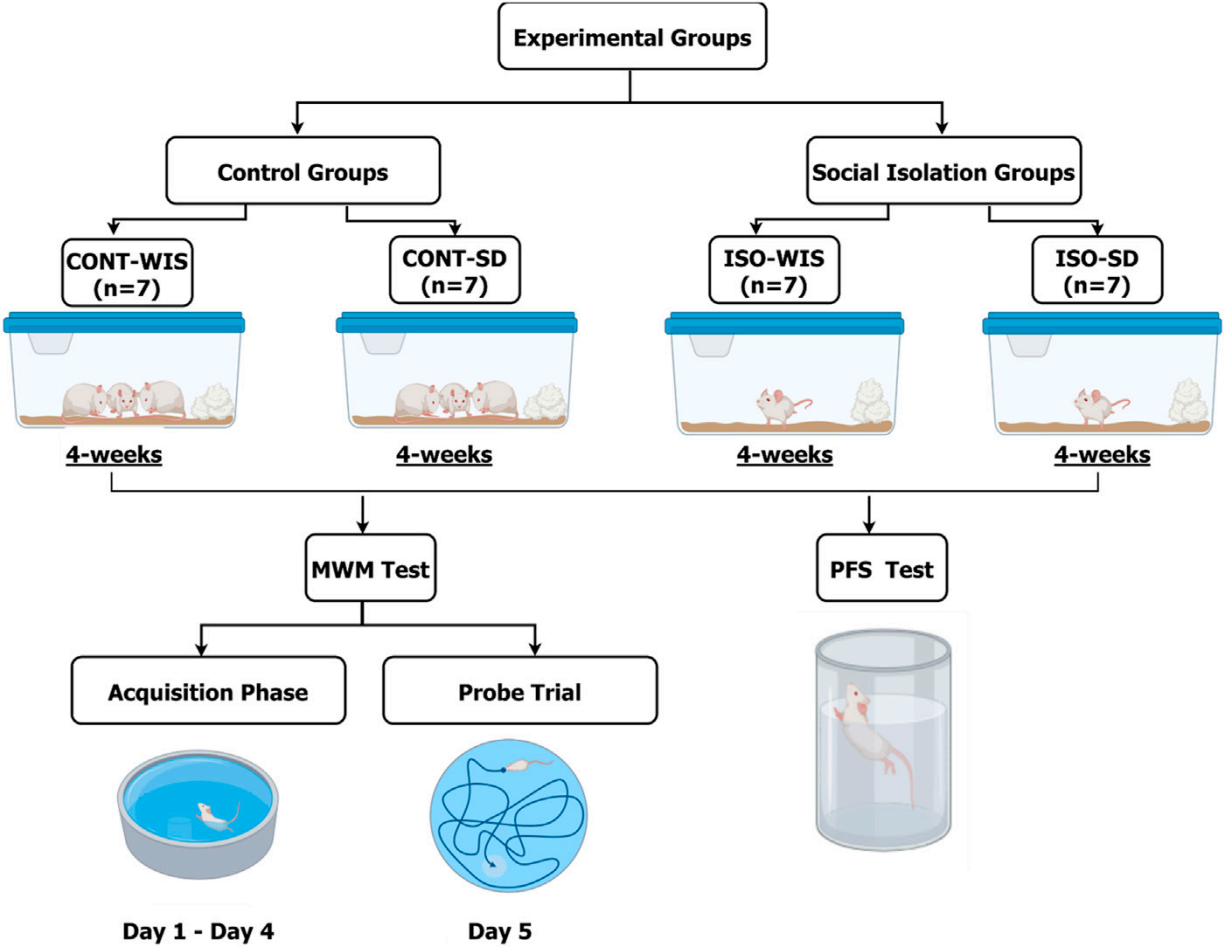
Experimental design of the study. CONT-WIS: Wistar control group, CONT-SD: Sprague Dawley control group, ISO-WIS: Wistar rats exposed to the social isolation, ISO-SD: Sprague Dawley rats exposed to the social isolation, MWM: Morris Water Maze. The rats were grouped as shown. CONT-WIS and CONT-SD are the control group of WIS and SD strains, respectively; ISO-WIS and ISO-SD are the corresponding social isolation groups. After 4 weeks, the MWM test was carried out on days 1–4 (acquisition phase) and on day 5 (probe trial). Created partially with BioRender.com.

The rats were divided into two groups. The control groups were kept in standard housing conditions, as shown above. After 4 weeks of social isolation, Morris Water Maze tests were performed for four consecutive days, first on the isolated rats, and then on the control group. The rats were placed in the laboratory 1 hour before the experiments started to allow them to acclimatize. The tests were initiated at 10 a.m. The control groups of each strain (WIS and SD) were named CONT-WIS (n = 10) and CONT-SD (n = 7), while social isolation groups were called ISO-WIS (n = 9) and ISO-SD (n = 8) (Figure 1).

### 2.3 Social isolation protocol

The rats in the ISO-WIS and ISO-SD groups were housed individually in enriched cages for 4 weeks. The rats in the control groups (CONT-WIS and CONT-SD) were housed in groups (n = 3–4/group). There was no physical contact between the isolated rats and their conspecifics. Due to the potential effects of handling on the results (Song et al., 2021), the researchers did not handle the rats before the experiments. The same person cleaned and changed the cages twice a week to reduce handling and interaction.

### 2.4 Behavioral tests

After the rats were acclimatized to the laboratory and researchers, the Morris Water Maze test was performed. Environmental cues were placed on the testing room’s walls. All tests were recorded with a video camera placed on the ceiling of the testing room. The images were transferred to a computer and analyzed with EthoVision XT 9 video tracking software.

#### 2.4.1 Morris water maze test

The study was conducted in a black circular pool (180 cm diameter, 60 cm depth) with water at a constant temperature (24°C–26°C). Visual cues, such as plus, circle, and rectangle signs were placed on the room’s walls, where the pool was located. The pool was divided into four hypothetical equal quadrants to decide where the animal should be released into the pool. A constant black platform (40 cm high, 2 cm below water) was placed in the middle of one quadrant.

The rats were tested twice daily over 4 days, and each session involved four releases from different quadrants. They swam for 90 s to locate the platform. Upon finding it, the rat waited on the platform for 30 s before being placed back into the pool from a different starting point. If the platform could not be found, the rats were put on the platform by the researcher for 30 s. Escape latency, the time taken to find the platform was recorded for each session, and daily averages were calculated.

During the probe trial to assess the memory on the fifth day, the platform was removed to examine the rats’ memory functions. During a single 90-s swimming session, the duration spent in the quadrant where the platform was initially positioned, the number of platform crossings, the distance covered, and swimming velocity were recorded.

#### 2.4.2 Porsolt forced swimming test

A transparent cylindrical Plexiglas tank (60 cm high, 20 cm in diameter) was employed to evaluate depression and learned helplessness. The tank was filled with water at 25°C, reaching a depth of 30 cm from surface level to prevent possible escape. A camera was placed above the cylinder to capture the test. The procedure involved placing a rat inside the tank and starting both a stopwatch and the video camera as soon as the rat entered the water. The durations for climbing, swimming and immobility exhibited by the rats were recorded over 2 days. The pre-test was conducted 2 days after Morris Water Maze. The initial day served as a pre-test, during which the animal remained in the water tank for 15 min, with its behavior being thoroughly documented using the Ethovision XT nine software. After each trial, the rats were carefully dried with a towel and a hairdryer before being returned to their respective cages. The next day the testing phase took place, with the animal placed in the tank again, this time for 5 min. The same parameters and measurement criteria were applied, and the rat was removed from the tank after the test and dried as described before.

### 2.5 Cortical tissue isolations

The day after the Porsolt test, the rats were decapitated under isoflurane anesthesia and their cortices were dissected in ice-cold saline (+4°C, 50 mL). The isolated cortex was used for trace element analysis by ICP-MS.

### 2.6 ICP-MS analysis of trace elements in rat cortices

#### 2.6.1 Sample pretreatment

The isolated cortex samples were weighed in acid-washed Eppendorf tubes and dried at 80 °C (drying oven) until constant weight. Then, the samples (approximately 1 mg) were transferred to 15 mL polypropylene tubes. Then 250 µL of high-purity HNO_3_ (≥69%, TraceSELECT, Fluka) and 50 µL of high-purity H_2_O_2_ (30%, Suprapur, Supelco) were added to digest the sample for 72 h at room temperature and 1 h at 60°C. After sample digestion, the volume was adjusted with ultrapure water and internal standards (IS) solution to a final volume of 10 mL. Sample blanks were obtained using the same procedure. The analytical quality was assessed using two certified reference materials (CRM) were used: Mussel tissue (ERM-CE278K) and Skimmed Milk Powder (ERM-BD151), both from the European Commission’s Joint Research Centre (JRC). These CRMs were pre-treated and analyzed using the same procedure as the samples. The obtained solutions were stored at 4°C until analysis.

#### 2.6.2 Trace element analysis

The samples were analyzed using inductively coupled plasma mass spectrometry (ICP-MS)with an iCAP Q instrument (Thermo Fisher Scientific), equipped with a Meinhard TQ + concentric quartz nebulizer, a Peltier-cooled high-purity quartz baffled cyclonic spray chamber, and a demountable quartz torch with a 2.5 mm i. d. quartz injector. The interface consisted of two Ni cones (sampler and skimmer). High-purity argon (99.9997%) was used as nebulizer and plasma gas. The sample solutions and calibration standards were introduced into the ICP-MS instrument using a CETAC ASX-520 autosampler (Teledyne CETAC Technologies). Before each run, the instrument was tuned for maximum sensitivity and signal stability to minimize the formation of oxides and double-charge ions. The main operational parameters of the ICP-MS were: nebulizer gas flow, 1.14 L/min; auxiliary gas flow, 0.79 L/min; plasma gas flow, 13.9 L/min; radiofrequency generator power, 1550 W; and dwell time, 1–10 m. The isotopes 7Li, 25Mg, 27Al, 31P, 43Ca, 52Cr, 55Mn, 57Fe, 59Co, 60Ni, 65Cu, 66Zn, 75As, 82Se, 85Rb, 88Sr, 111Cd, 121Sb, 133Cs, 137Ba, 205 TL and 208 Pb were measured for analytical determination and the isotopes 6Li, 45Sc, 71Ga, 89Y, 103Rh, 193Ir and 209Bi were monitored as IS (Azevedo et al., 2023).

### 2.7 Statistical analysis

Data analysis was conducted using the statistical software package SPSS version 22. Graphs were generated in the Graphpad Prism 10.2.1. A one-way analysis of variance (ANOVA) was employed to examine the effects of the time spent in the platform area (duration), the number of platform crossings (frequency), the average speed in the pool (velocity) and the total distance (distance) in the probe trial (CONT-WIS, ISO-WIS, CONT-SD and ISO-SD). Prior to conducting the ANOVA, assumptions of normality, homogeneity of variances were assessed through visual inspection of histograms, and Levene’s test, respectively. Subsequently, to test specific hypotheses regarding the linear trend across group means, a linear contrast analysis was performed. Three contrasts were created to assess linear trends across the groups. Trace element analysis was performed with One-Way ANOVA and the data is represented as “F (DFn, DFd) = Fvalue, *p*-value” with *p* < 0.05 significant difference. The analysis of immobility time was performed with repeated-measures analysis of variance (ANOVA) followed by *post hoc* Dunnett test, designed with two factors, “time” and “treatment,” followed by the Dunnett test, was used.

## 3 Results

### 3.1 Morris water maze

#### 3.1.1 Time spent to find the platform (escape latency) over the course of four days

**FIGURE 2:**
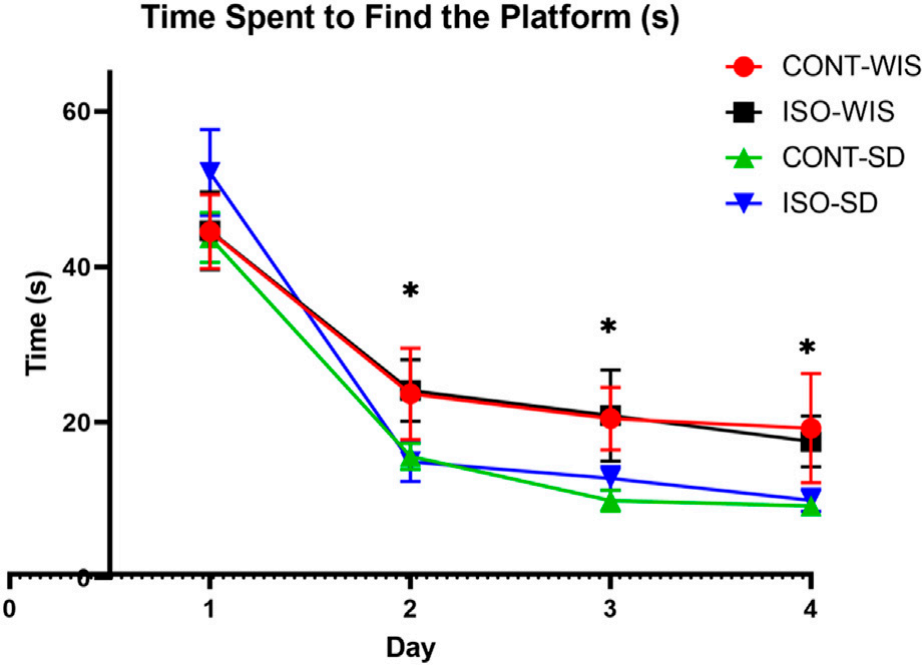
Time spent by the rats to find the platform (seconds). All data are expressed as mean ± SEM. *p < 0.05 indicates a significant difference between the time points of first and second day. No significant differences were found between the groups.

Briefly, all rats learned to find the platform, as shown by the significant decrease in escape latency compared to the first day within each group. There was no statistically significant difference in the time spent to find the platform between all groups on day 1. However, significant differences were observed between the time points, but no significant results were observed with the effect of strain or social [F (2,252, 67,55) = 71,82, *p* < 0.05] significant differences of first day of the second, third and fourth days (Figure 2).

#### 3.1.2 Probe trial

**FIGURE 3:**
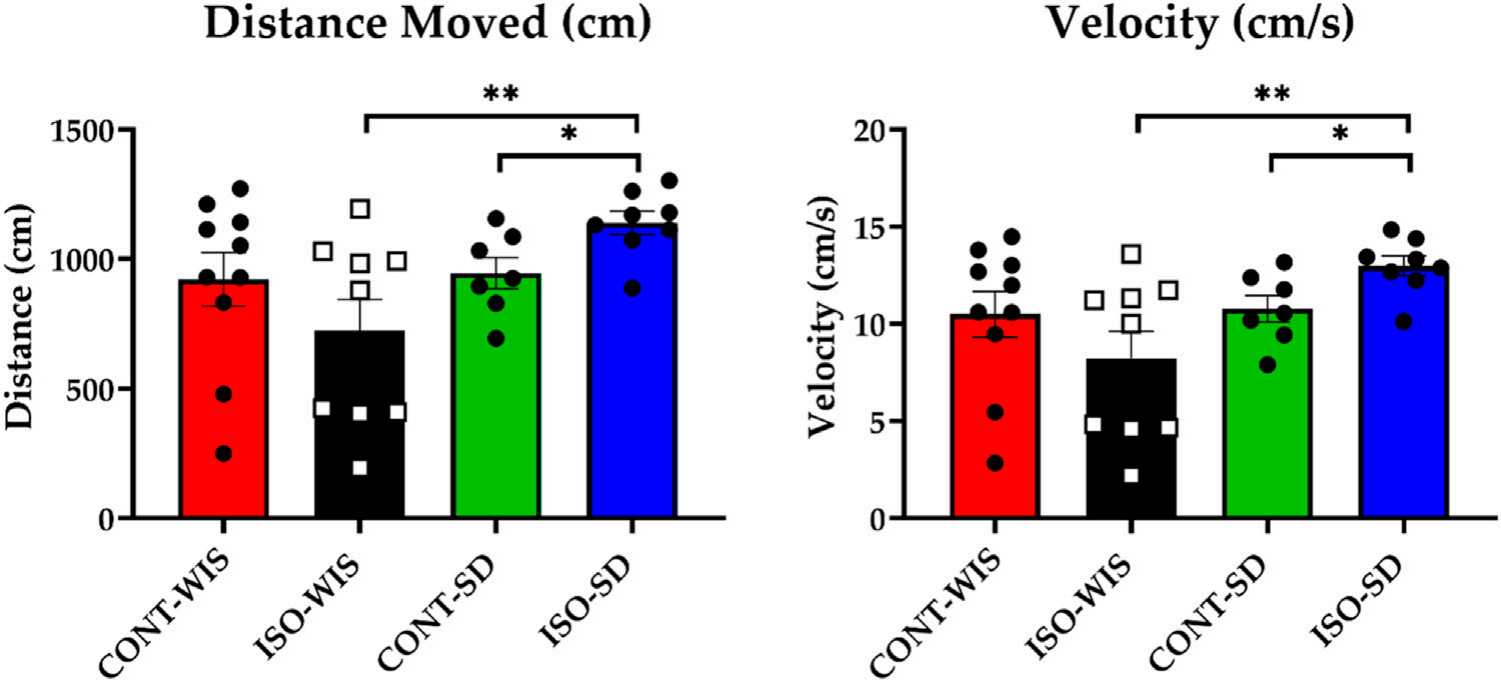
Results of the probe trial: Distance Travelled (cm) and Velocity (cm/s) for each group. All data are expressed as mean ± SEM. *p < 0.05, **p < 0.01 denote significant differences. The distance travelled was compared based on the effect of social isolation or strain differences between the groups of CONT-WIS vs. ISO-WIS, CONT-SD vs. ISO-SD, CONT-WIS vs. CONT-SD, and ISO-WIS vs. ISO-SD. Velocity was compared based on the effect of social isolation on compared based on the effect of social isolation or strain differences between the groups of CONT-WIS vs. ISO-WIS, CONT-SD vs. ISO-SD, CONT-WIS vs. CONT-SD, and ISO-WIS vs. ISO-SD.

In the probe trial, memory performance was assessed by time spent in the target quadrant, number of platform crossings, average velocity, and total distance swam. Statistical differences were found in the distance travelled (t = 0.205, *p* < 0.05) and velocity (t = 0.205, *p* < 0.05) between ISO-WIS and ISO-SD (Figures 3D–H Supplementary Figure S4) and between the CONT-SD and ISO-SD (Figures 3B, F).

**FIGURE 4:**
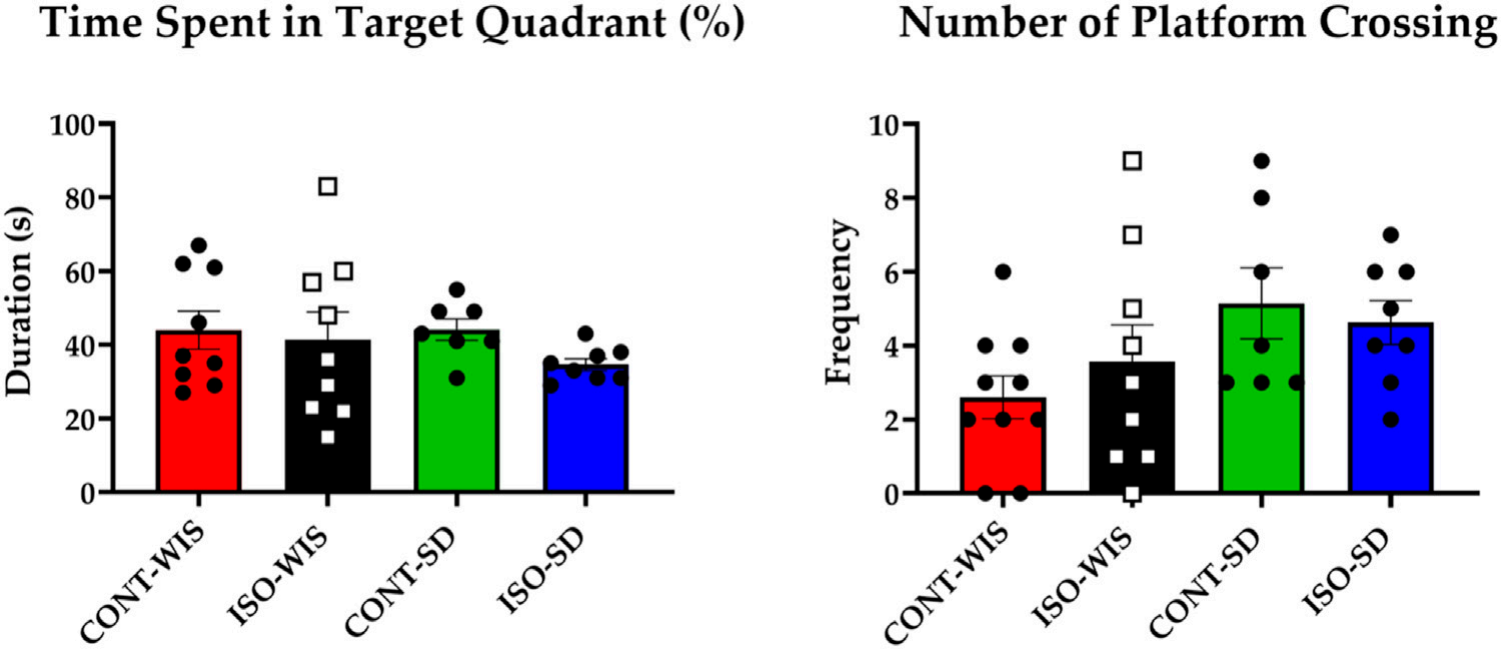
Probe trial parameter results: Time Spent in the Target Quadrant (Duration; s) and the Number of Platform Crossings (Frequency) for each group. All data are expressed as mean ± SEM. Time spent in the target quadrant and the number of platform crossings based on the effect of social isolation or strain differences between the groups of CONT-WIS vs. ISO-WIS, CONT-SD vs. ISO-SD, CONT-WIS vs. CONT-SD, and ISO-WIS vs. ISO-SD. Velocity was compared based on the effect of social isolation on compared based on the effect of social isolation or strain differences between the groups of CONT-WIS vs. ISO-WIS, CONT-SD vs. ISO-SD, CONT-WIS vs. CONT-SD, and ISO-WIS vs. ISO-SD.

No significant linear contrasts were observed for groups that the time spent in target quadrant and the number of platform crossings were compared (*p* > 0.05; Figure 4).

### 3.2 Porsolt forced swimming test

**FIGURE 5:**
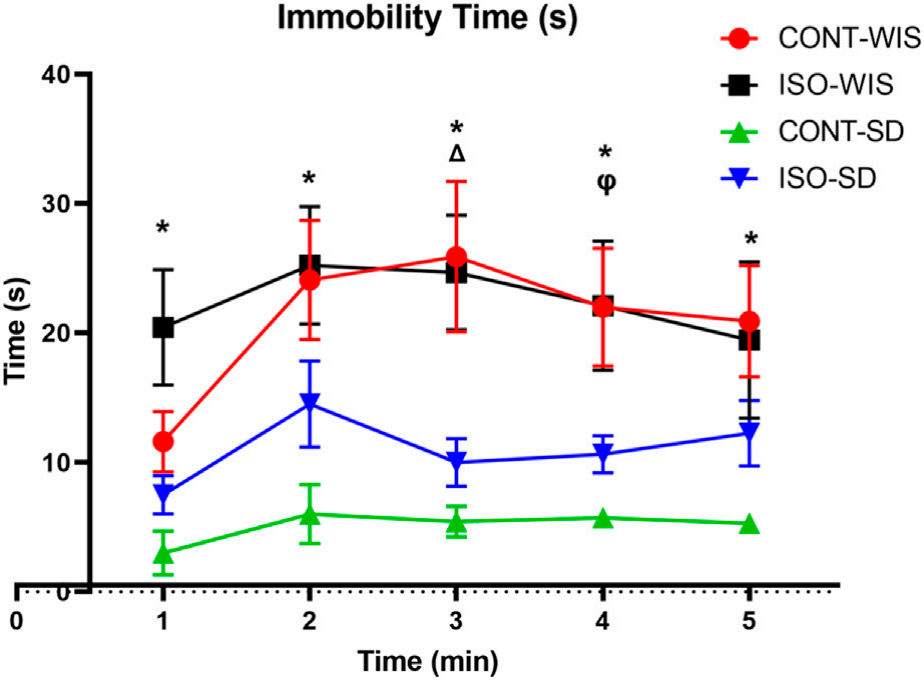
Time of Immobility (seconds). All data are expressed as mean ± SEM. *p < 0.05, indicate significant difference. The significant differences between groups are denoted as follows: *p < 0.05, CONT-WIS and CONT-SD; ^Δ^ *p* < 0.05, ISO-WIS and ISO-SD; ^ᵩ^ *p* < 0.05, CONT-SD and ISO-SD.

The Porsolt Forced Swimming test results were compared between all groups. Two-Way Anova results showed a significant difference between groups in immobility time (*p* < 0.05) at 1, 2, 3, 4 and 5 min. There was also a significant difference in isolated animals (*p* < 0.005). The difference in the immobility time between the ISO-WIS and CONT-WIS, CONT-SD and CONT-WIS as well as CONT-SD and ISO-SD groups were statistically significant [strain or social isolation effect; F (Van der Borght et al., 2005; Lukkes et al., 2009) = 6,583, *p* < 0.05](Figure 5).

### 3.3 Trace element levels in the cortex

**FIGURE 6:**
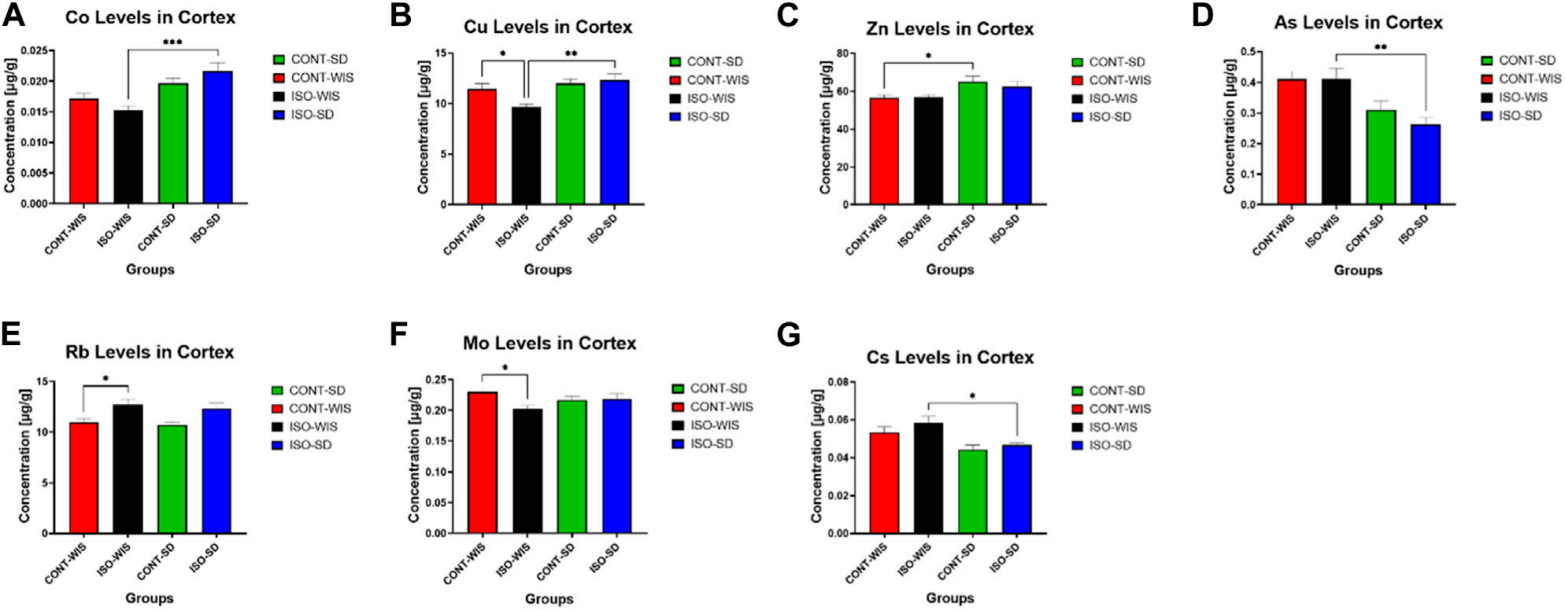
Cortical trace element levels. **(A)** Co levels in the cortex in all groups, **(B)** Cu levels in the cortex in all groups, **(C)** Zn levels in the cortex in all groups, **(D)** As levels in the cortex in all groups, **(E)** Rb levels in the cortex in all groups, **(F)** Mo levels in the cortex in all groups, **(G)** Cs levels in the cortex in all groups. All data are expressed as mean ± SEM. The significant differences between groups are denoted as follows: *p < 0.05, Cu levels between the CONT-WIS and ISO-WIS, Zn levels between CONT-WIS and CONT-SD, Rb levels between CONT-WIS and ISO-WIS, Mo levels between CONT-WIS and ISO-WIS, Cs levels between ISO-WIS and ISO-SD groups. **p < 0.01, Cu levels between the ISO-WIS and ISO-SD, As levels between ISO-WIS and ISO-SD. ***p < 0.001, Co levels between the ISO-WIS and ISO-SD.

The trace element levels (As, Ba, Ca, Co, Cr, Cs, Fe, Hg, Li, Mg, Mn, Mo, P, Rb, Se, Sr, Tl, Zn) were measured in the rat cortices from all groups (See Supplementary Material for other trace element levels for all elements: ^7^Li, ^25^Mg, ^27^Al, ^31^P, ^43^Ca, ^52^Cr, ^55^Mn, ^57^Fe, ^59^Co, ^60^Ni, ^65^Cu, ^66^Zn, ^75^As, ^82^Se, ^85^Rb, ^88^Sr, ^111^Cd, ^121^Sb, ^133^Cs, ^137^Ba, ^205^TL and ^208^Pb). The levels of Co, Cu, Zn, As, Rb, Mo and Cs were significantly different between some groups. Co and Cu levels were significantly higher in the ISO-SD group than the ISO-WIS group (F (Rex et al., 2004; Lukkes et al., 2009) = 1.284; *p* = 0.3014; F (Rex et al., 2004; Lukkes et al., 2009) = 6.776; *p* = 0.0017; respectively, Figure 6). Whereas Cs and As levels were significantly lower in the ISO-SD group compared to the ISO-WIS group (F (Rex et al., 2004; Lukkes et al., 2009) = 5.421; *p* = 0.0052; F (Rex et al., 2004; Lukkes et al., 2009) = 6.616; *p* = 0.0019; respectively, Figure 6). After social isolation, Cu and Mo levels were lower (F (Rex et al., 2004; Lukkes et al., 2009) = 3.191; *p* = 0.0409) in the CONT-WIS group than the ISO-WIS group (F (Rex et al., 2004; Lukkes et al., 2009) = 4.750; *p* = 0.0093), while Rb levels were higher. In the naïve strains were compared, it was observed that Zn levels were lower in the WIS group that in the SD rats (F (Weiss et al., 2000; Lukkes et al., 2009) = 4.114; *p* = 0.0173; Figure 6).

## 4 Discussion

Until now, a direct and comprehensive assessment of trace elements in the brain, such as a comparative analysis between two different rat strains following social isolation has not been done yet. Therefore, our objective was to discern potential associations between trace element levels, strain responses to social isolation, and subsequent impacts on depressive behavior and memory functions. Our findings revealed differences in trace element levels between two rat strains subjected to social isolation. Specifically, Co and Cu levels were elevated, while As and Cs levels were reduced in the socially isolated SD group compared to the WIS group.

Our findings showed that, compared to the first day, the time spent finding the platform gradually decreased for all groups. In summary: (Liu et al., 2010): although the distance to find the platform in the probe trial was not affected by isolation in the WIS groups, it had a drastic effect on the SD groups; (Cacioppo and Hawkley, 2009); the velocity of the isolated SD was higher than the SD control and isolated WIS groups. No differences were found between CONT-SD and CONT-WIS groups; (Lukkes et al., 2009); isolated SD rats found the platform faster than control SD rats, but no other differences were found regarding velocity; (Amiri et al., 2015); there were significant differences in the cortical trace element levels between groups. Specifically, within the ISO-SD group, unlike the ISO-WIS group, the Co and Cu levels were higher, while the Cs and As levels were lower; (Filipović et al., 2011); isolation led to decreased levels of Cu and Mo, but increased the Rb levels in WIS rats. Comparing trace element levels in naïve groups of different strains, Zn levels were lower in the WIS group than in the SD rats; (Martinovic et al., 2014); the CONT-SD and ISO-SD groups outperformed the CONT-WIS group in locating the platform over 4 days although statistically not significant.

Our results suggest that social isolation may impact ISO-SD rats more. Existing literature on the behavioral consequences of social isolation in various rat strains varies widely. Specifically, one study documented a substantial decline in pre-pulse inhibition among SD rats compared to WIS rats after 12 weeks of isolation (Weiss et al., 2000). In our research, ISO-SD rats had increased velocity and distance moved that might show it takes more effort to find the platform, which might indicate a potential memory deficit. Our results also revealed that isolation elicited a comparatively more discernible impact on SD than WIS rats. Previous reports have suggested that environmental conditions do not significantly impact the social behavior of SD rats, while they affect WIS rats indicating that WIS rats generally tend to be more anxious (Rex et al., 2004).

The Morris Water Maze test is used for evaluating memory functions and spatial learning mainly the time spent in the target quadrant and the number of platform crossings. Total distance travelled and the average swimming speed of the rats was also included as it was shown in prior studies that anxiety and age affected these parameters as well, as well as to understand the thigmotactic behaviors (Belviranli et al., 2012). Repeated testing was conducted for 4 days to assess spatial learning, and a probe trial day to assess the reference memory performance (Morris, 1984). In both control and isolated groups, SD rats performed better than WIS rats in locating the platform over 4 days as seen in the graphs although there was no statistical significance. This might suggest that SD rats might possess better spatial learning and memory ability than the WIS rats, but more studies are needed to confirm this. Adult SD rats have been previously shown to perform better than WIS rats in autoshaping, lever press response, maze testing (Andrews et al., 1995), and the latency to find the maze (Inostroza et al., 2011). However, another study comparing WIS and SD rats reported contradictory results. Although both strains performed equally during training, WIS rats exhibited better cueing results in the Morris Water Maze test (Van der Borght et al., 2005). Which might be in line with the increased velocity and distance taken by SD rats in the probe trial. Another study comparing three rat strains revealed that SD rats are superior to spontaneous hypertensive rats. A genetic model of attention deficit hyperactivity disorder also localized the platform in the maze more rapidly (Cao et al., 2013). Furthermore, compared with WIS Kyoto rats and spontaneous hypertensive rats, SD rats exhibited elevated docosahexaenoic acid levels in their hippocampus, prefrontal cortex, and corpus striatum, and reduced docosahexaenoic acid levels in the corpus callosum, parietal lobe, and temporal lobe. Our findings are consistent with the studies on aged rats.

Using the Porsolt forced swimming test we evaluated depressive-like behavior in rats using immobility time. We observed significant differences between CONT-WIS and CONT-SD groups throughout the experiments. The immobility time for CONT-WIS rats was longer than that of CONT-SD rats for each time period, suggesting that WIS rats exhibited more anxiety than naturally middle-aged SD rats. Although also a statistically significant difference was observed between isolated WIS/SD rats, it is difficult to interpret this due to the baseline difference in anxiety. The duration of immobility of WIS rats was shown to be longer than that of SD rats, which might be because WIS rats have more dopamine receptors (Zamudio et al., 2005). Furthermore, the ISO-WIS group showed longer immobility time than the CONT-WIS group in the first minute, but without statistical significance. A study on 15-week-old rats showed no significant difference between isolated and control WIS rats regarding immobility time (Hall et al., 1998). As a limitation of our study, we initially conducted behavioral tests with the isolated group while many animals were in the groups. This ensured that the time spent in the cages during social isolation was evenly distributed, involving 17 animals first, considering constraints related to daylight hours and the duration of behavioral tests.

While direct associations are challenging to establish, there is evidence of altered levels of trace elements that may indicate a potential association with social isolation and its outcomes. For instance, Zn levels were higher in the SD control group, aligning with previous research associating Zn with stress-related responses. Zn deficiency or social isolation alone induces anxious or depressive symptoms (Mitsuya et al., 2015). In previous findings, socially-isolated rats showed reduced serum Zn levels but increased Cu levels in the prefrontal cortex of SD rats (Famitafreshi and Karimian, 2016). In our study, Zn was also initially higher in the control group of SD. Similarly, the activity of total superoxide dismutase, including Cu/Zn superoxide dismutase (CuZnSOD) and catalase was increased in the cerebral cortex of WIS rats exposed to social isolation (Pejic et al., 2016). Another study reports increased activity of CuZnSOD in socially isolated rats (Pajović et al., 2006; Stanisavljevic et al., 2017). These increases in the Cu and Zn may be related to the increases in the need for the catalase activities. Zn was also associated with depressive-like and anxiety-related behavior (Takeda et al., 2007; Watanabe et al., 2010). The effects of Zn were studied in the forced swimming (Porsolt’s) test in mice. Zn (ZnSO4) at a dose of 30 mg/kg (but not at a dose of 10 mg/kg), similar to imipramine (30 mg/kg), reduced the immobility time in that test. Moreover, Zn at both doses reduced the locomotor activity. The obtained results indicate that Zn induces an antidepressant-like effect in the forced swimming test. Since Zn reduces locomotor activity, this antidepressant-like effect is not related to the alteration of general activity (Kroczka et al., 2000). In our study, not many differences were found due to social isolation. Still, SD rats had higher intrinsic levels of Zn, which may be associated with an intrinsic protection mechanism against possible stressors. In contrast, SD rats appear more prone to stress-related immobility behaviors.

The antidepressant, psychostimulant, and nootropic effects of several major and trace elements including KCl, RbNO3, and magnesium sulphate were studied on models of behavioral despair using Porsolt and conditioned passive avoidance tests. This preparation was found to shorten the immobilization time in the Porsolt test and promote retention of the conditioned passive avoidance. The most pronounced psychostimulant effect of the substance was observed in a dose-dependent manner (Afanasieva et al., 2013). Other trace elements have been associated with neurological symptoms or irritability such as Mo, Rb, exposure to As and elevated Co levels (Rex et al., 2004; Tower, 2010; Kyi-Tha-Thu et al., 2023). We have found elevated Rb levels only in socially isolated WIS rats, thus supporting the anticipated association between their breed disposition and elevated Rb levels under isolation conditions (Rex et al., 2004). We have found decreased Mo levels in the ISO-WIS group. Animal studies have shown reduced exploratory behavior and a potential decline in passive avoidance learning in subjects exposed to higher doses of Co (Czarnota et al., 1998). It has also been demonstrated that top‐down control of passive coping behavior by the medial prefrontal cortex can collaborate with the bottom‐up action of glucocorticoids to improve memory consolidation of the immobility response. In this regard, the immobility response can be attributed to the accumulation of passive avoidance behavior (Lingg et al., 2020). In line with these findings, isolated SD rats in our study showed greater immobility compared to isolated WIS rats in the Porsolt forced swimming test, as well as increased Co levels, which may be a possible association of correlated neurobehavioral changes. In male rats, chronic exposure to As resulted in anxiety- and depression-like behaviors along with memory impairment. These effects were observed through reduced time spent in open areas (elevated plus maze), increased immobility time (forced swimming test), and decreased performance in spatial memory tasks (Morris water maze test) (Samad et al., 2021). In the course of our research, elevated As levels were noted in ISO-WIS rats in comparison to ISO-SD rats. This observation aligns with the inherently anxious disposition of WIS rats and previous research indicating the anxiogenic effects of As. Furthermore, a disparity emerged in the Porsolt forced swimming test between ISO-WIS and ISO-SD rats only in the third minute, with ISO-WIS rats displaying significantly greater immobility compared to ISO-SD. While consistent with earlier findings on the impact of As, it is noteworthy that social isolation exerts a more pronounced influence on the SD strain. One study associated Cs with impaired learning and spatial memory and increased anxiety (Bellés et al., 2016). Further, regarding anxiety, which is expected to be higher in WIS rats, our results show that CONT-WIS had higher levels of Cs compared to CONT-SD, consistent with previous findings.

In summary, distinct patterns were evident in both trace element analysis and behavioral tests. The comprehensive findings indicate elevated Co and Cu levels, coupled with decreased Cs and As levels in socially isolated SD rats compared to WIS rats. Furthermore, isolation may have exerted varying influences on Zn, Mo, and Rb levels within each strain. This divergence in trace element profiles is perceived as offering valuable insights into factors contributing to resilience. Moreover, the present results hold promise for identifying novel targets for the treatment and dietary prevention of depressive and mental disorders during stressful periods in the future. The study emphasizes the strain-specific responses to isolation, providing an in-depth view of the neurobehavioral effects of trace elements and contributing valuable information to the comprehension of behavioral consequences in socially isolated rats. Other factors also must be considered to draw direct associations between these associations.

## 5 Conclusion

Studies suggest that strain differences might be the underlying reason behind the conflicting results in any behavioral paradigm. A strain’s performance in one task does not reliably predict its performance on another. Our findings indicate that SD rats are more sensitive to social isolation as shown in previous studies. Therefore, using SD rats to study the effects of stress and isolation is more plausible. Furthermore, we emphasized strain-specific responses to isolation and the association of specific neurobehavioral effects with trace elements. Our results might contribute to the future on designing a balanced diet to prevent neurobehavioral abnormalities due to social isolation.

## Data Availability

The original contributions presented in the study are included in the article/Supplementary Material, further inquiries can be directed to the corresponding author.

## Supplementary material

Supplementary Figure 1 Cortical trace element levels. Co, Mg, P, Ca, Cr, Mn, Fe, Co, Cu levels in the cortex in all groups. All data are expressed as mean ± SEM. The significant differences between groups are denoted as follows: **p* < 0.05, Cu levels between the CONT-WIS and ISO-WIS, ***p* < 0.01, Cu levels between the ISO-WIS and ISO-SD, ****p* < 0.001, Co levels between the ISO-WIS and ISO-SD.

Supplementary Figure 2 Cortical trace element levels. Zn, As, Se, Rb, Sr, Mo, Cs, Ba, Hg levels in the cortex in all groups. All data are expressed as mean ± SEM. The significant differences between groups are denoted as follows: **p* < 0.05, Zn levels between the CONT-WIS and CONT-SD, Rb levels between the CONT-WIS and ISO-WIS, Mo levels between CONT-WIS and ISO-WIS, Cs levels ISO-WIS and ISO-SD, ***p* < 0.01, As levels between the ISO-WIS and ISO-SD.
