# Supplementary figures and images for: Relationships between trace elements and cognitive and depressive behaviors in sprague dawley and wistar albino rats
Source: Front Pharmacol. 2024 Apr 2;15:1367469. doi: 10.3389/fphar.2024.1367469 (PMC11018905; doi:10.3389/fphar.2024.1367469)

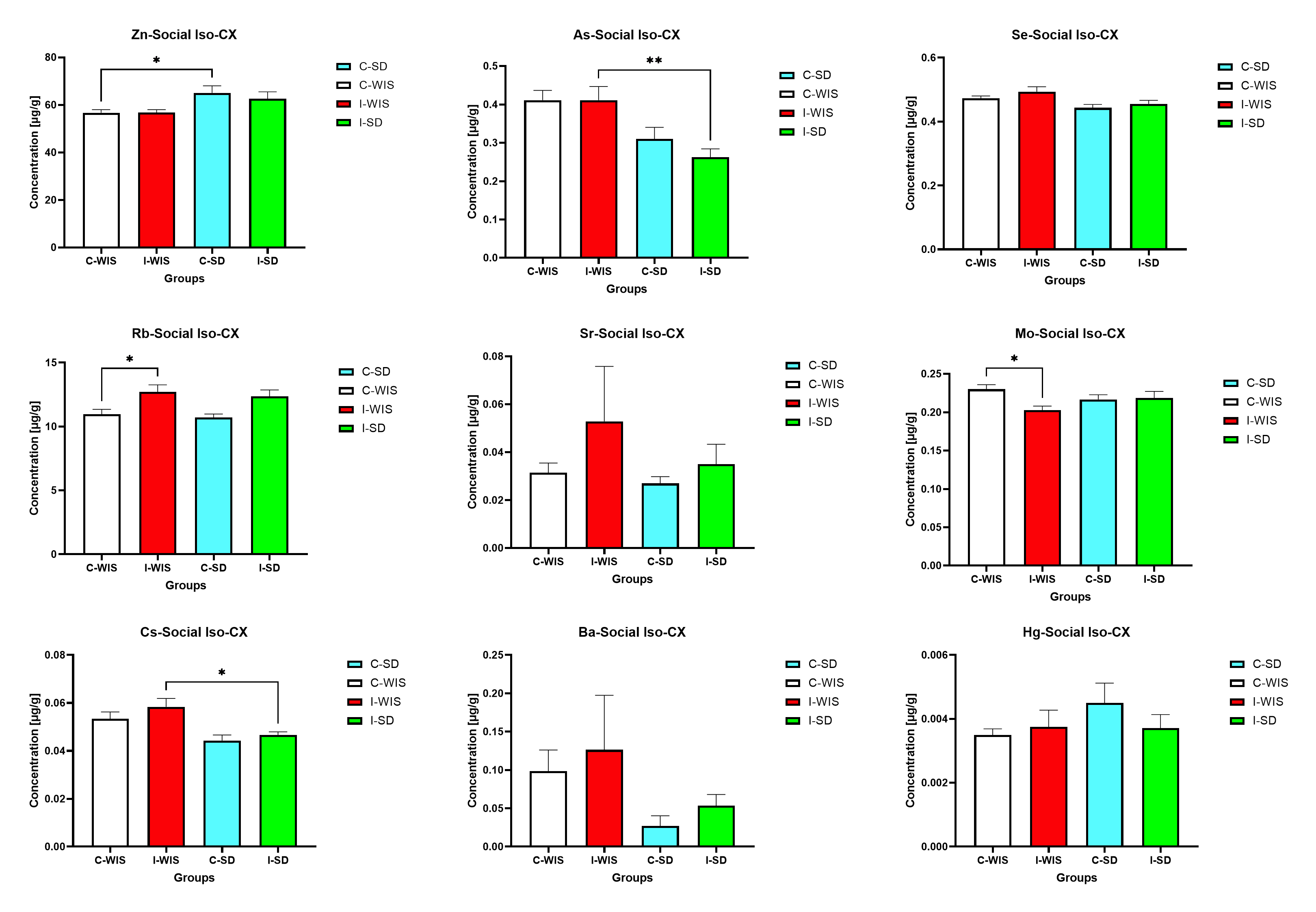

Supplement: Supplementary file 1 [file Image2.TIF]

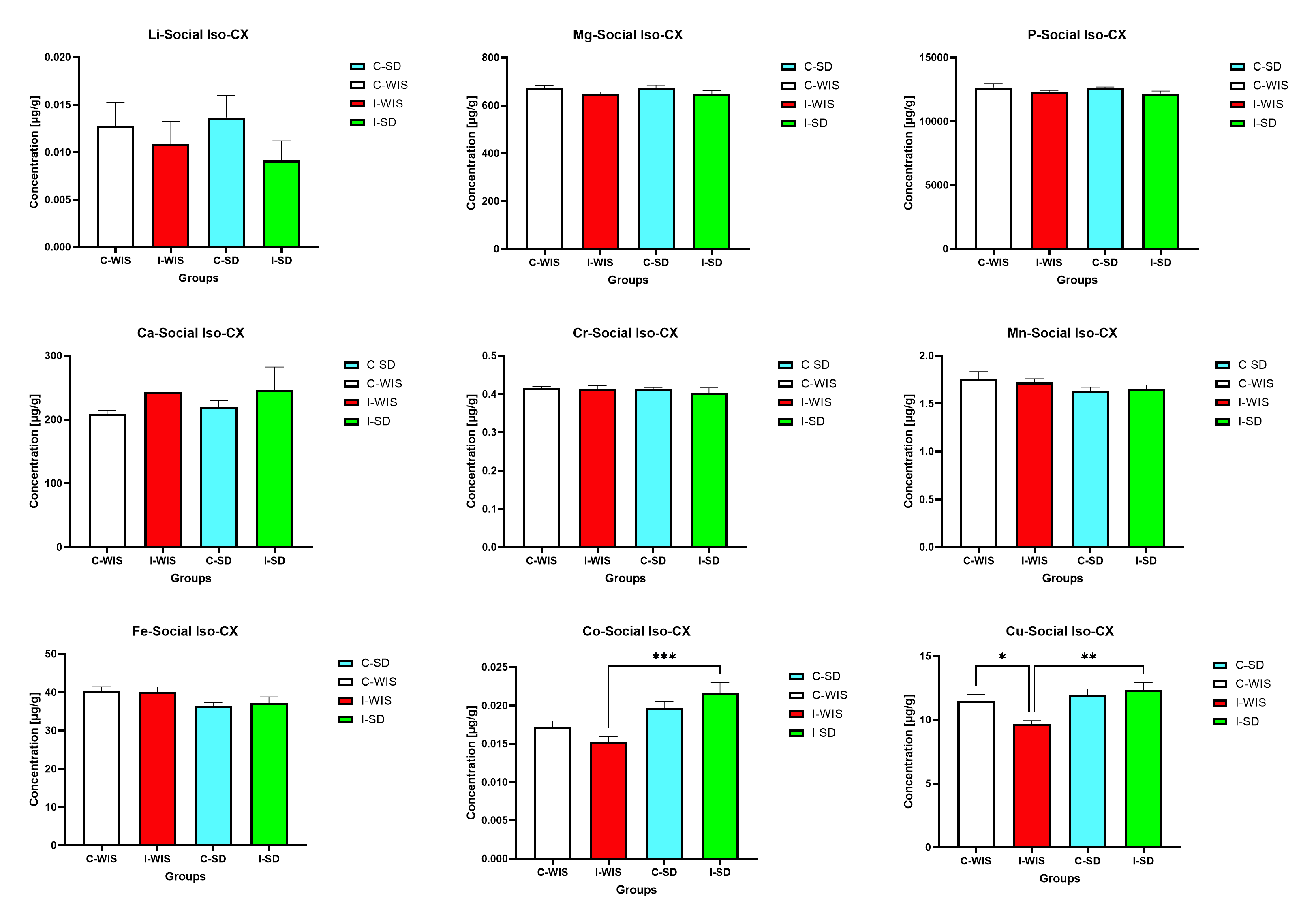

Supplement: Supplementary file 2 [file Image1.TIF]
